# Characteristics of out-of-hospital cardiac arrest patients in Riyadh province, Saudi Arabia: a cross-sectional study

**DOI:** 10.3389/fcvm.2023.1192795

**Published:** 2023-05-22

**Authors:** Alyaman Almiro, Osamah AlQassab, Rasmieh Alzeidan, Abdulaziz Saad Binhaddab, Ahmad M. Alkhorisi, Hani A Almalki, Muhannad Abdulaziz Ghouthalsayd, Tarek Kashour, Ahmed Hersi, Wael Alqarawi

**Affiliations:** ^1^ College of Medicine, Alfaisal University, Riyadh, Saudi Arabia; ^2^ Department of Cardiac Sciences, College of Medicine, King Saud University, Riyadh, Saudi Arabia; ^3^ Saudi Red Crescent Authority, Riyadh, Saudi Arabia; ^4^ Operation Center, Public Health Agency, Saudi Ministry of Health, Riyadh, Saudi Arabia; ^5^ University of Ottawa Heart Institute, University of Ottawa, Ottawa, ON, Canada

**Keywords:** out-of-hospital cardiac arrest (OHCA), cardiac arrest, cardiopulmonary resuscitation (CPR), emergency medical services (EMS), Riyadh, Saudi Arabia

## Abstract

**Introduction:**

Little work has been done on out-of-hospital cardiac arrest (OHCA) in Saudi Arabia. Our goal is to report the characteristics of OHCA patients and predictors of bystander cardiopulmonary resuscitation (CPR).

**Materials and methods:**

This cross-sectional study utilized data from the Saudi Red Crescent Authority (SRCA), a governmental emergency medical service (EMS). A standardized data collection form based on the “Utstein-style” guidelines was developed. Data were retrieved from the electronic patient care reports that SRCA providers fill out for every case. OHCA cases that were attended by SRCA in Riyadh province between June 1st, 2020 and May 31st, 2021 were included. Multivariate regression analysis was performed to assess independent predictors of bystander CPR.

**Results:**

A total of 1,023 OHCA cases were included. The mean age was 57.2 (±22.6). 95.7% (979/1,023) of cases were adults and 65.2% (667/1,023) were males. Home was the most common location of OHCA [784/1,011 (77.5%)]. The initial recorded rhythm was shockable in 131/742 (17.7%). The EMS mean response time was 15.9 min (±11.1). Bystander CPR was performed in 130/1,023 (12.7%) and was more commonly performed in children as compared to adults [12/44 (27.3%) vs. 118/979 (12.1%), *p* = 0.003]. Independent predictors of bystander CPR were being a child (OR = 3.26, 95% CI [1.21–8.82], *p* = 0.02) and having OHCA in a healthcare institution (OR = 6.35, 95% CI [2.15–18.72], *p* = 0.001).

**Conclusion:**

Our study reported the characteristics of OHCA cases in Saudi Arabia using EMS data. We observed young age at presentation, low rates of bystander CPR, and long response time. These characteristics are distinctly different from other countries and call for urgent attention to OHCA care in Saudi Arabia. Lastly, being a child and having OHCA in a healthcare institution were found to be independent predictors of bystander CPR.

## Introduction

Out-of-hospital cardiac arrest (OHCA) is a major public health issue worldwide. Emergency medical services (EMS)-treated OHCA incidence rates range from 19.2 to 150.1 per 100,000 person-years globally (1). Following OHCA, survival to discharge ranges from 0.6% to 25% in different parts of the world (1). Moreover, in the USA, it was found that only 8.5% of adults with EMS-treated nontraumatic OHCA survived with good neurological function (2).

Regional variations in epidemiological characteristics and outcomes of OHCA patients are well-reported in the literature (1, 3). These variations are possibly due to differences in definitions of OHCA used (1). However, unique demographic, medical, healthcare system, and EMS characteristics of these regions also play a role. Subsequently, each region must generate its own data to better characterize its OHCA population. This would be done as the first step towards exploring reasons for regional variation as well as identifying areas that are lacking in that particular region so that future interventions can be implemented to improve and optimize the care provided to OHCA patients.

Only two studies have examined the characteristics of OHCA patients in Saudi Arabia and were limited by small sample sizes and selection bias (4, 5). In 1999, Conroy and Jolin reported the characteristics of 66 OHCA cases who presented to a tertiary hospital over a 7-year period (4). A more recent study was conducted in 2015 and included 96 adult OHCA cases who presented to a university hospital in Riyadh (5). Both studies were single-center studies and included a small number of OHCA cases, which limits their generalizability. Therefore, the OHCA population in Saudi Arabia is yet to be appropriately characterized. This lack of OHCA characterization is hindering the improvement and optimization of OHCA care in Saudi Arabia. As such, we sought to describe the characteristics of OHCA patients attended by EMS in Riyadh province, Saudi Arabia, and identify predictors of bystander cardiopulmonary resuscitation (CPR).

## Materials and methods

This is a cross-sectional study of OHCA patients who were attended by EMS in Riyadh province, Saudi Arabia.

### Source of data and study population

Data for this study was obtained from the Saudi Red Crescent Authority (SRCA) records. SRCA is the governmental EMS that serves all regions of Saudi Arabia (including Riyadh province) free of charge for all residents. Following each attended incident, SRCA providers fill out a standardized electronic patient care report, which was the source of data used in this study. All cases that had out-of-hospital cardiac arrest and occurred in Riyadh province between June 1st, 2020 and May 31st, 2021 were included. Cases that did not receive CPR were excluded (i.e., pronounced dead upon arrival of EMS).

### Variables

We developed a data collection form that follows the “Utstein-style” guidelines and definitions for reporting OHCA (6). It contained the following variables: age, sex, nationality, location, incident type, initial recorded rhythm, bystander CPR, incident month, incident time, and response time. Locations were categorized into home, public setting, workplace, and healthcare institution which was defined as an institution where out-patient healthcare is provided. Incident type is the chief complaint recorded in the electronic patient care report. Incident time is the time when SRCA was called, and response time is the time difference in minutes between incident time and time of management initiation by SRCA.

### Statistical analysis

Continuous variables were presented as means (±standard deviation) and categorical variables were presented as numbers (percentages). The age groups used in this study are defined as follows: child (<18), and adult (≥18). Incident month and time were categorized into 4 equal quartiles. Chi-square test, Fisher's exact test, or Student's *T*-test were used to compare groups. A *p* value <0.05 was considered to be statistically significant. Multivariate logistic regression was performed to assess independent predictors of bystander CPR. For the purpose of the logistic regression analysis, incident type was categorized into “traumatic” vs. “non-traumatic”. All clinically-significant variables were included in the model. Analyses were performed using SAS (version 9.4, The SAS Institute, USA).

### Ethical approval

This study was approved by the institutional review board at King Khalid University Hospital.

## Results

### Total population

**Figure 1:**
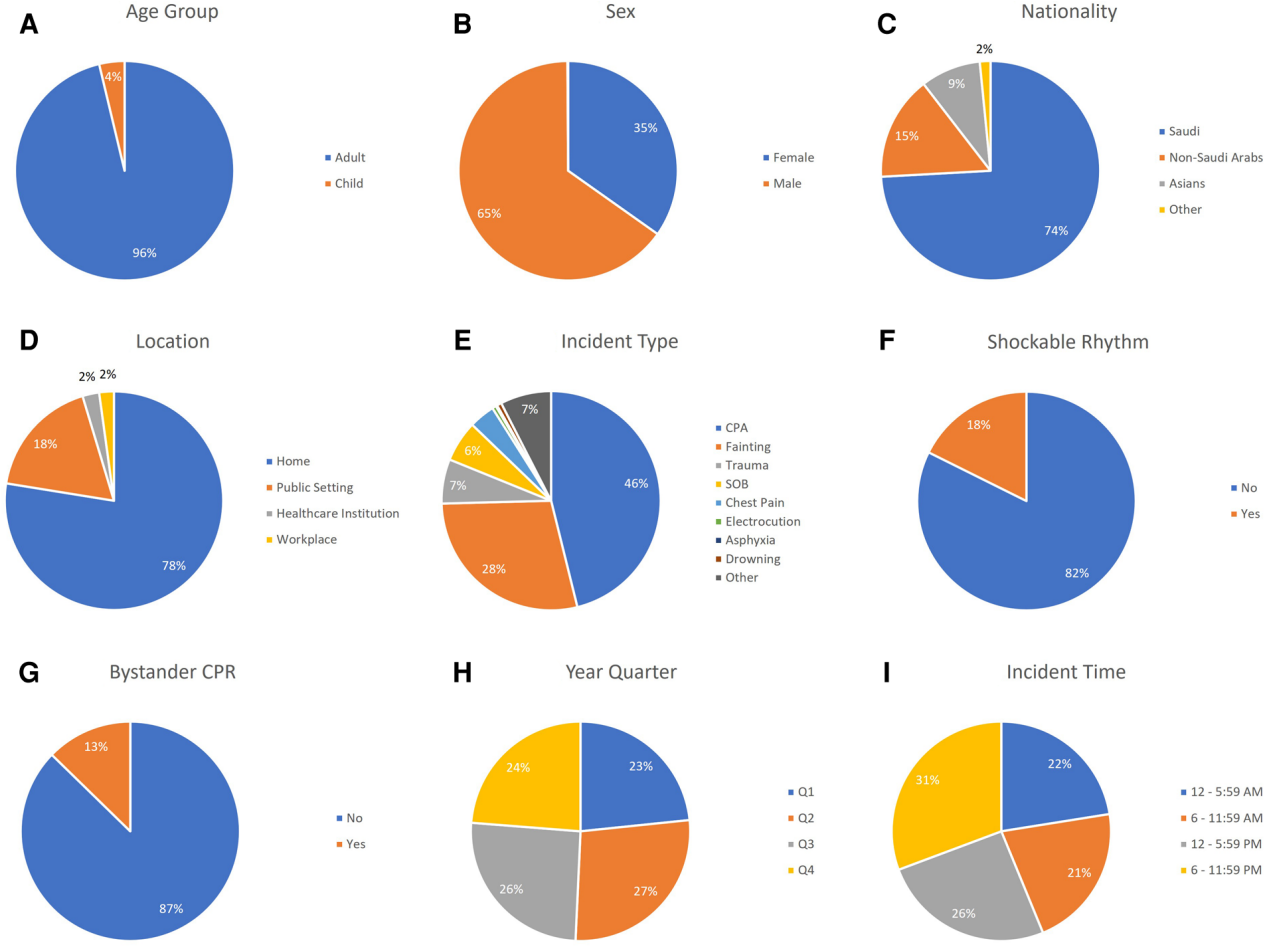
Baseline characteristics of OHCA patients in Riyadh province, Saudi Arabia. (**A**) Shows age group distribution. (**B**) Shows sex distribution. (**C**) Shows nationality distribution. (**D**) Shows location distribution. (**E**) Shows incident type distribution. (**F**) Shows shockable rhythm distribution. (**G**) Shows bystander CPR distribution. (**H**) Shows year quarter distribution. (**I**) Shows incident time distribution. CPA, cardiopulmonary arrest; SOB, shortness of breath; CPR, cardiopulmonary resuscitation; Q1, quarter 1; Q2, quarter 2; Q3, quarter 3; Q4, quarter 4.

A total of 1,023 patients with OHCA were included in this study. OHCA patients had a mean age of 57.2 (±22.6) years and 65.2% were males (667/1,023). Home was the most common location of OHCA [784/1,011 (77.5%)]. Bystander CPR was performed in 130/1,023 (12.7%) of cases. Incident time was evenly distributed among the 4 time quadrants. The most common nationality was Saudi [659/889 (74.1%)] followed by non-Saudi Arab [137/889 (15.4%)] and Asian [79/889 (8.9%)]. Table 1 and Figure 1 show baseline characteristics of the total population in our study.

**Table 1:** Baseline characteristics of out-of-hospital cardiac arrest patients.

Variable	Findings (*N* = 1,023)
Mean age	57.2 ± 22.6
Age group (adults)	979 (95.7%)
Sex (male)	667 (65.2%)
Nationality	
– Saudi	659/889 (74.1%)
– Non-Saudi Arabs	137/889 (15.4%)
– Asians	79/889 (8.9%)
– Other	14/889 (1.6%)
Location	
– Home	784/1,011 (77.5%)
– Public setting	180/1,011 (17.8%)
– Healthcare institution	25/1,011 (2.5%)
– Workplace	22/1,011 (2.2%)
Incident type	
– CPA	472 (46.1%)
– Fainting	291 (28.4%)
– Trauma	67 (6.5%)
– SOB	62 (6.1%)
– Chest pain	39 (3.8%)
– Electrocution	6 (0.6%)
– Asphyxia	2 (0.2%)
– Drowning	7 (0.7%)
– Other	77 (7.5%)
Shockable rhythm	131/742 (17.7%)
Bystander CPR	130 (12.7%)
Year quarter	
– Q1	239 (23.4%)
– Q2	280 (27.4%)
– Q3	261 (25.5%)
– Q4	243 (23.8%)
Incident time	
– 12–5:59 AM	230 (22.5%)
– 6–11:59 AM	218 (21.3%)
– 12–5:59 PM	261 (25.5%)
– 6–11:59 PM	314 (30.7%)
Mean response time (min)	15.9 ± 11.1

Data are presented as means (±standard deviation) or numbers (percentages). CPA, cardiopulmonary arrest; SOB, shortness of breath; CPR, cardiopulmonary resuscitation.

### Adult vs. pediatric patients

We included 979 adults (95.7%) and 44 children (4.3%) in our study. Home was the most common location of OHCA for both. However, children had higher percentages of OHCA in public settings and healthcare institutions as compared to adults [14 (31.8%) and 5 (11.4%) for children vs. 166/967 (17.2%) and 20/967 (2.1%) for adults, respectively, *p* < 0.0001]. Drowning and trauma were more common incident types in children than adults [5 (11.4%) and 8 (18.2%) for children vs. 2 (0.2%) and 59 (6%) for adults, respectively, *p* < 0.0001]. Response time was significantly shorter for children as compared with adults (13.4 ± 7.9 vs. 16 ± 11.2, *p* = 0.043). Table 2 shows baseline characteristics of OHCA cases as stratified by adult and pediatric age groups.

**Table 2:** Baseline characteristics of out-of-hospital cardiac arrest patients, as stratified by age groups.

Variable	Adult (*N* = 979)	Child (*N* = 44)	*p* value
Mean age	59.6 ± 20.2	5.2 ± 4.4	–
Sex (male)	641 (65.5%)	26 (59.1%)	0.385
Nationality			
– Saudi	631/854 (73.9%)	28/35 (80%)	0.418
– Non-Saudi	223/854 (26.1%)	7/35 (20%)	
Location			
– Home	759/967 (78.5%)	25 (56.8%)	0.002
– Public setting	166/967 (17.2%)	14 (31.8%)	
– Other	42/967 (4.3%)	5 (11.4%)	
Incident type			
– CPA	453 (46.3%)	19 (43.2%)	0.006
– Fainting	284 (29%)	7 (15.9%)	
– Trauma	59 (6%)	8 (18.2%)	
– Other	183 (18.7%)	10 (22.7%)	
Shockable rhythm	129/713 (18.1%)	2/29 (6.9%)	0.141
Bystander CPR	118 (12.1%)	12 (27.3%)	0.003
Year quarter			
– Q1	230 (23.5%)	9 (20.5%)	0.914
– Q2	266 (27.2%)	14 (31.8%)	
– Q3	250 (25.5%)	11 (25%)	
– Q4	233 (23.8%)	10 (22.7%)	
Incident time			
– 12–5:59 AM	225 (23%)	5 (11.4%)	0.002
– 6–11:59 AM	212 (21.7%)	6 (13.6%)	
– 12–5:59 PM	253 (25.8%)	8 (18.2%)	
– 6–11:59 PM	289 (29.5%)	25 (56.8%)	
Mean response time (min)	16 ± 11.2	13.4 ± 7.9	0.043

Data are presented as means (±standard deviation) or numbers (percentages). CPA, cardiopulmonary arrest; SOB, shortness of breath; CPR, cardiopulmonary resuscitation.

### Shockable vs. non-shockable rhythm

Table 3 summarizes baseline characteristics of OHCA cases as stratified by the initial recorded rhythm. The two groups had mostly comparable characteristics. However, the shockable rhythm group had a higher percentage of males (74.8% vs. 65.1%, *p* = 0.033) as compared to the non-shockable rhythm group.

**Table 3:** Baseline characteristics of out-of-hospital cardiac arrest patients, as stratified by the initial recorded rhythm.

Variable	Shockable (*N* = 131)	Non-shockable (*N* = 611)	*p* value*
Mean age	60.1 ± 18.3	57.5 ± 22.7	0.169
Age group (adult)	129 (98.5%)	584 (95.6%)	0.121
Sex (male)	98 (74.8%)	398 (65.1%)	0.033
Nationality			
– Saudi	90/122 (73.8%)	375/515 (72.8%)	0.831
– Non-Saudi	32/122 (26.2%)	140/515 (27.2%)	
Location			
– Home	96 (73.3%)	487/602 (80.9%)	0.085
– Public setting	25 (19.1%)	91/602 (15.1%)	
– Other	10 (7.6%)	24/602 (4%)	
Incident type			
– CPA	66 (50.4%)	306 (50.1%)	0.223
– Fainting	41 (31.3%)	173 (28.3%)	
– Trauma	1 (0.8%)	28 (4.6%)	
– Other	23 (17.6%)	104 (17%)	
Bystander CPR	20 (15.3%)	85 (13.9%)	0.686
Year quarter			
– Q1	31 (23.7%)	106 (17.3%)	0.003
– Q2	35 (26.7%)	165 (27%)	
– Q3	25 (19.1%)	208 (34%)	
– Q4	40 (30.5%)	132 (21.6%)	
Incident time			
– 12–5:59 AM	29 (22.1%)	131 (21.4%)	0.97
– 6–11:59 AM	28 (21.4%)	132 (21.6%)	
– 12–5:59 PM	31 (23.7%)	156 (25.5%)	
– 6–11:59 PM	43 (32.8%)	192 (31.4%)	
Mean response time (min)	16.1 ± 12.4	15.5 ± 10.5	0.662

Data are presented as means (±standard deviation) or numbers (percentages). CPA, cardiopulmonary arrest; SOB, shortness of breath; CPR, cardiopulmonary resuscitation.

### Bystander CPR

Supplementary Table S1 shows baseline characteristics of OHCA cases as stratified by the performance of bystander CPR. A higher proportion of children received bystander CPR as compared to adults [12/44 (27.3%) vs. 118/979 (12.1%), *p* = 0.003]. Home was the most common location in both groups; however, the bystander CPR group was more likely to be at a healthcare institution and less likely to be in a public setting [13/129 (10.1%) and 11/129 (8.5%) for bystander CPR group vs. 12/882 (1.4%) and 169/882 (19.2%) for no bystander CPR group, respectively, *p* < 0.0001]. Table 4 shows the results of univariate and multivariate logistic regression analysis of independent predictors of bystander CPR. Independent predictors of bystander CPR were being a child (OR = 3.26, 95% CI [1.21–8.82], *p* = 0.02) and having OHCA at a healthcare institution (OR = 6.35, 95% CI [2.15–18.72], *p* = 0.001).

**Table 4:** Univariate and multivariate logistic regression analysis of independent predictors of bystander cardiopulmonary resuscitation in out-of-hospital cardiac arrest patients.

Univariate			Multivariate		
Variable	OR 95% CI	*p* value	Variable	OR 95% CI	*p* value
Age group			Age group		
– Adult	REF	–	– Adult	REF	–
– Child	2.74 (1.37–5.46)	0.004	– Child	3.26 (1.21–8.82)	0.02
Sex			Sex		
– Male	REF	–	– Male	REF	–
– Female	1.03 (0.7–1.51)	0.881	– Female	1.23 (0.75–2.01)	0.408
Nationality			Nationality		
– Saudi	REF	–	– Saudi	REF	–
– Non-Saudi	1.36 (0.84–2.19)	0.21	– Non-Saudi	1.54 (0.85–2.78)	0.155
Location type			Location type		
– Home	REF	–	– Home	REF	–
– Public setting	0.45 (0.23–0.85)	0.014	– Public setting	0.87 (0.35–2.11)	0.751
– Healthcare institution	7.41 (3.29–16.69)	<0.001	– Healthcare institution	6.35 (2.15–18.72)	0.001
– Workplace	2.01 (0.73–5.57)	0.179	– Workplace	2.46 (0.46–13.11)	0.291
Incident type			Incident type		
– Traumatic	REF	–	– Traumatic	REF	–
– Non-traumatic	5.02 (1.22–20.77)	0.026	– Non-traumatic	2.97 (0.33–26.39)	0.329
Shockable rhythm			Shockable rhythm		
– Yes	REF	–	– Yes	REF	–
– No	0.9 (0.53–1.52)	0.686	– No	0.9 (0.49–1.65)	0.736
Year quarter			Year quarter		
– Q1	REF	–	– Q1	REF	–
– Q2	2.21 (1.27–3.85)	0.005	– Q2	1.94 (0.95–3.95)	0.068
– Q3	2.16 (1.23–3.79)	0.007	– Q3	1.5 (0.74–3.06)	0.261
– Q4	0.98 (0.51–1.88)	0.956	– Q4	0.81 (0.35–1.86)	0.612
Incident time			Incident time		
– 12–5:59 AM	REF	–	– 12–5:59 AM	REF	–
– 6–11:59 AM	0.94 (0.54–1.64)	0.835	– 6–11:59 AM	0.81 (0.39–1.65)	0.558
– 12–5:59 PM	1.07 (0.63–1.8)	0.808	– 12–5:59 PM	1.18 (0.62–2.26)	0.621
– 6–11:59 PM	0.89 (0.53–1.49)	0.659	– 6–11:59 PM	0.84 (0.44–1.62)	0.609

## Discussion

Our study is the first to examine the characteristics of OHCA and predictors of bystander CPR in Saudi Arabia using EMS data. We observed young age at presentation, low rate of bystander CPR, and long response time when compared with published literature from other countries (Supplementary Table S2). We hope that our findings will ignite interest and guide further research aiming at improving the suboptimal care of OHCA in Saudi Arabia.

The mean age of adult OHCA cases in our study was 59.6, which was similar to previous smaller studies performed in Saudi Arabia and the Gulf region (Supplementary Table S2) (4, 5, 7) However, this is significantly younger than the average age reported by western countries such as the USA (63.7) and England (68.6) (8, 9). The likely reason for this apparent younger age at presentation in Saudi Arabia is the young overall age of the Saudi population as compared to other countries. However, further research needs to ascertain if the high prevalence of cardiovascular risk factors such as diabetes mellitus and hypertension plays a role (10). This young age at presentation highlights the paramount importance of improving survival of OHCA in Saudi Arabia given the potential years of life lost and its effect on the community in general.

A small percentage of patients received bystander CPR in our study (12.7%). This is clearly in contrast to reported rates from other countries such as the USA (34.4%), Singapore (45.7%), England (55.2%), Victoria-Australia (58.5%), Norway (73%), and the Netherlands (81.2%) (8, 9, 11–13). It is well known that bystander CPR is associated with significant improvement in survival from OHCA, as this has been demonstrated by multiple studies over the years (9, 14–18). Therefore, it should be a priority target to improve the survival of OHCA patients especially since it is an inexpensive and readily available modifiable factor. Indeed, several nationwide initiatives aimed at increasing resuscitation by bystanders in Denmark were associated with doubling the rate of bystander CPR (from 21.1% in 2001 to 44.9% in 2010), which also were associated with improved overall survival (19).

Low bystander CPR rates have been explored previously in the literature (20–23). Multiple factors were found to be contributing such as lack of training, lack of confidence, fear of causing harm, fear of legal liability, and fear of acquiring infections (20–23). It has been hypothesized that middle eastern societies' set of values may provide a cultural barrier, particularly in the case of female patients (7). However, our study does not support this hypothesis given that the rate of bystander CPR was similar in both sexes [12.9% (46/356) in females as compared to 12.6% (84/667) in males]. Further studies need to examine causes of low bystander CPR rates in order to develop specific interventions aiming at improving it.

In our study, we found that being a child was an independent predictor of receiving bystander CPR (OR = 3.26, *p* = 0.02). This might be explained by the fact that children who are at risk of cardiac arrest are likely to have parents who are trained in performing CPR. The other independent predictor was the location of the arrest being in a healthcare institution (OR = 6.35, *p* = 0.001), which is expected as personnel in such settings are trained in CPR and are more confident in performing it.

A major finding of our study is the long EMS response time, which is double the acceptable international resuscitation benchmark of 8 min. One needs to study OHCA in other provinces in Saudi Arabia to determine whether this is specific to Riyadh province or a general feature of Saudi Arabia. This is especially important since Riyadh is known for its traffic congestion, large area, and rapid expansion which might render achieving the optimal EMS response time more difficult. EMS response time has been demonstrated to be inversely proportional to survival in OHCA in various studies (24–26). Smart solutions to achieve the acceptable response time have been proposed, such as the use of unmanned aerial vehicles (UAV), commonly known as drones. It was demonstrated by multiple studies that using UAVs can lead to faster delivery of automated external defibrillators as compared to EMS (27–31). In Stockholm County in Sweden, Claesson et al. performed a simulation study to test the effectiveness of UAVs' delivery of automated external defibrillators to OHCA cases and to determine whether their use can decrease response time (27). They used coordinates of previous OHCA cases in Stockholm County to determine the optimal placement of UAVs in urban and rural areas. Using Geographic Information System (GIS) models, they predicted that UAVs would arrive faster than EMS in 32% of urban OHCA cases (with a mean time saved of 1.5 min) and in 93% of rural OHCA cases (with a mean time saved of 19 min). Utilization of such innovations could potentially improve response time and subsequently OHCA survival, especially in an area such as Riyadh province where conventional methods can be challenging.

Our study has several limitations. First, we did not have access to in-hospital outcomes to determine the survival rate. Second, only limited data were available in the electronic patient care reports. Last, patients with OHCA who were brought to hospitals by means other than SRCA and those who did not receive CPR were not represented. Nonetheless, we believe our study provided important findings that can guide future research and initial interventional plans to improve the care of OHCA in Saudi Arabia.

## Conclusion

This study identified key characteristics of OHCA in Riyadh province, Saudi Arabia. Young age at presentation, low rate of bystander CPR, and long EMS response time are the main characteristics that significantly differ from international standards. Additionally, we found being a child and having OHCA at a healthcare institution to be independent predictors of bystander CPR. More research is needed to identify factors that influence these characteristics and interventions that could improve them.

## Data Availability

The original contributions presented in the study are included in the article/**Supplementary Material**, further inquiries can be directed to the corresponding author.
