# Supplementary material for: Characteristics of out-of-hospital cardiac arrest patients in Riyadh province, Saudi Arabia: a cross-sectional study
Source: Front Cardiovasc Med. 2023 May 22;10:1192795. doi: 10.3389/fcvm.2023.1192795 (PMC10239974; doi:10.3389/fcvm.2023.1192795)
Supplement: Supplementary file 1 [file Table1.docx]

**Supplementary Table 1:** Baseline characteristics of out-of-hospital cardiac arrest patients, as stratified by the performance of bystander cardiopulmonary resuscitation.

| Variable | Bystander CPR Performed (N=130) | Bystander CPR Not Performed (N=893) | P value |
| --- | --- | --- | --- |
| Mean Age | 54.5 ± 25.8 | 57.6 ± 22.1 | 0.191 |
| Age Group (adult) | 118 (90.8%) | 861 (96.4%) | 0.003 |
| Sex (male) | 84 (64.6%) | 583 (65.3%) | 0.881 |
| Nationality   - Saudi - Non-Saudi | 90/114 (78.9%)  24/114 (21.1%) | 569/775 (73.4%)  206/775 (26.6%) | 0.208 |
| Location   - Home - Public Setting - Healthcare Institution - Workplace | 100/129 (77.5%)  11/129 (8.5%)  13/129 (10.1%)  5/129 (3.9%) | 684/882 (77.6%)  169/882 (19.2%)  12/882 (1.4%)  17/882 (1.9%) | <0.0001 |
| Incident Type   - CPA - Fainting - Trauma - Other | 82 (63.1%)  36 (27.7%)  2 (1.5%)  10 (7.7%) | 390 (43.7%)  255 (28.6%)  65 (7.3%)  183 (20.5%) | <0.0001 |
| Shockable Rhythm | 20/105 (19%) | 111/637 (17.4%) | 0.686 |
| Year Quarter   - Q1 - Q2 - Q3 - Q4 | 20 (15.4%)  47 (36.2%)  43 (33.1%)  20 (15.4%) | 219 (24.5%)  233 (26.1%)  218 (24.4%)  223 (25%) | 0.001 |
| Incident Time   - 12 - 5:59 AM - 6 - 11:59 AM - 12 - 5:59 PM - 6 - 11:59 PM | 30 (23.1%)  27 (20.8%)  36 (27.7%)  37 (28.5%) | 200 (22.4%)  191 (21.4%)  225 (25.2%)  277 (31%) | 0.905 |
| Mean Response Time (min) | 15.6 ± 11.2 | 15.9 ± 11.1 | 0.767 |
| Data are presented as means (± standard deviation) or numbers (percentages).  CPR: cardiopulmonary resuscitation. CPA: cardiopulmonary arrest. SOB: shortness of breath. | | | |
