# Supplementary material for: Characteristics of out-of-hospital cardiac arrest patients in Riyadh province, Saudi Arabia: a cross-sectional study
Source: Front Cardiovasc Med. 2023 May 22;10:1192795. doi: 10.3389/fcvm.2023.1192795 (PMC10239974; doi:10.3389/fcvm.2023.1192795)
Supplement: Supplementary file 2 [file Table2.docx]

**Supplementary Table 2:** Comparing baseline characteristics of out-of-hospital cardiac arrest patients between this study and other national and international studies.

| Study Name/Year | Country | Mean Age | Sex (male) | Location | Incident Type | Shockable Rhythm | Bystander CPR | Comments |
| --- | --- | --- | --- | --- | --- | --- | --- | --- |
| This Study | Saudi Arabia | 57.2 ± 22.6 | 65.2% | Home: 77.5%  Public Setting: 17.8%  Healthcare Institution: 2.5%  Workplace: 2.2% | CPA: 46.1%  Fainting: 28.4%  Trauma: 6.5%  SOB: 6.1%  Chest Pain: 3.8%  Electrocution: 0.6%  Asphyxia: 0.2%  Drowning: 0.7%  Other: 7. 5% | 17.7% | 12.7% | Includes cases of all ages |
| Conroy and Jolin (4)  1999 | Saudi Arabia | Adults: 55.4  Children: 5.3 | 57.3% | - | In adults:  Cardiac (29/39, 74.4%)  Noncardiac (10/39, 25.6%) | Adults (7/39, 17.9%) | Adults (2/39, 5.1%)  Children (0/27, 0%) | Includes cases of all ages |
| Bin Salleeh et al. (5)  2015 | Saudi Arabia | 58.9 | 62.5% | Home: 74%  Public Setting: 14.6% Rehabilitation Center: 4.2%  Other 7.2% | Cardiac: 82.3% Trauma: 12.5% Respiratory: 3.1% Other: 2.1% | Asystole: 94.8%  VT: 3.1%  Other: 2.1% | 20.8% | Includes cases of adults (18 years or more) only |
| Alqahtani et al. (7)  2019 | United Arab Emirates | 50 ± 22.3 | 77% | Home: 67.5%  Public Setting: 22.5%  Workplace: 6.7%  Healthcare Center: 2.7% | - | 12.5% | 30.7% | Includes cases of all ages |
| Hawkes et al. (8)  2017 | England | 68.6 ± 19.6 | 58.7% | Home: 60.8%  Non-home: 12.2%  Unknown: 27% | Cardiac: 60.9%  Trauma: 2.6%  Asphyxia: 2.1%  Other: 8.9% | 20.6% | 55.2% | Includes cases of all ages |
| Girotra et al. (9)  2016 | United States of America | 63.7 ± 16.2 | 63.6% | Home: 81.8%  Public Setting: 18.2% | - | 24.9% | 34.4% | Includes cases of adults (18 years or more) only |
| Lim et al. (11)  2020 | Singapore | 66.7 ± 16.5 | 65% | Home: 72.3%  Public Setting: 17.2  Aged Care: 3.7%  Other: 6.8% | Cardiac: 69.8%  Trauma: 3.3%  Respiratory/Medical: 5.4%  Other: 21.5% | 17.7% | 45.7% | Includes cases of adults (20 years or more) only |
| Lim et al. (11)  2020 | Victoria | 64.6 ± 17.7 | 69.2% | Home: 66.9%  Public Setting: 19.7%  Aged Care: 6.7%  Other: 6.7% | Cardiac: 74.8%  Trauma: 7.1%  Respiratory/Medical: 3.8%  Other: 14.3% | 30.3% | 58.5% | Includes cases of adults (20 years or more) only |
| CPA: cardiopulmonary arrest. SOB: shortness of breath. VT: ventricular tachycardia. | | | | | | | |  |
